# CORRELATES OF PHYSICIAN TRUST: DEMOGRAPHIC FACTORS AND EXPERIENCES OF MEDICAL CARE DISCRIMINATION

**DOI:** 10.1093/geroni/igad104.3248

**Published:** 2023-12-21

**Authors:** Lindsey Mundy, Suzanne Judd, Olivio Clay, Virginia Howard, Raegan Durant, Michael Crowe

**Affiliations:** University of Alabama at Birmingham, Birmingham, Alabama, United States; University of Alabama at Birmingham, Birmingham, Alabama, United States; University of Alabama at Birmingham, Birmingham, Alabama, United States; University of Alabama at Birmingham, Birmingham, Alabama, United States; University of Alabama at Birmingham, Birmingham, Alabama, United States; University of Alabama at Birmingham, Birmingham, Alabama, United States

## Abstract

Lack of trust between older adults and their physicians may decrease adherence to medication or limit disclosure of information to healthcare providers. Trust may be shaped in part by demographic factors as well as previous experiences of discrimination in healthcare. We hypothesized that more experiences of discrimination in a medical care setting would be negatively associated with general physician trust. We used cross-sectional data from a follow-up visit (2013-2016) of the national REasons for Geographic and Racial Differences in Stroke (REGARDS) longitudinal cohort study. Our sample included participants who completed an 11-item general physician trust measure (score range=11-55) and a four-point item about discrimination in medical care settings (n=8,235, age 52-98, 31% Black, 45% men). We examined two sequential linear regression models with trust as the outcome, with the first model including demographic factors and the second adding discrimination in a medical setting. Female gender (β=-0.09, p<.05), Black race (β=-0.03, p<.05), and higher level of education (β=-0.8, p<.05) were each related to lower trust. Higher income (β=0.04, p<.05) and older age (β=0.12, p<.05) were associated with higher trust. Perceived discrimination had a negative association with physician trust (β=-0.14, p<.05) and the relationship between Black race and trust reversed direction and was no longer statistically significant (β=0.20, p=.298) with discrimination in the model. The potential impact of previous discrimination experiences in a medical care setting on physician trust should be considered when providing healthcare services to diverse populations of older adults.

